# Extending the Definition of Computational Talent

**DOI:** 10.3390/bs16081278

**Published:** 2026-07-27

**Authors:** Marcos Román-González, Ana Vidal-Fernández

**Affiliations:** 1 Faculty of Education, National University of Distance Education (UNED), 28040 Madrid, Spain; mroman@edu.uned.es; 2 International Doctoral School of the UNED (EIDUNED), National University of Distance Education (UNED), 28015 Madrid, Spain

**Keywords:** computational thinking, computational talent, computer science education, programming, artificial intelligence, quantum education

## Abstract

Recent theoretical work has conceptualized computational thinking (CT) as a dynamic psychological construct that evolves in parallel with Computer Science (CS). From this perspective, a 3-stage CT framework has been proposed to reflect how the construct has evolved and is being expanded due to recent advancements in the CS field. More specifically, this extended framework comprises the following stages: ‘rule-based’ CT (1.0), linked to procedural programming and deterministic logic; ‘data-driven’ CT (2.0), influenced by advancements in artificial intelligence (AI) and machine learning (ML) and incorporating inductive logic and probabilistic reasoning; and ‘quantum’ CT (3.0), the emerging stage, associated with quantum computing (QC) and embracing a holistic philosophical paradigm. Within this broader perspective, the related psychological construct, namely computational talent, has been initially defined as the ability to progress from ‘visual block-based’ procedural programming environments to ‘text-based’ ones. Building on this rationale, the present theoretical essay pursues two main objectives: (i) to delve deeper into the 3-stage CT framework, using a tripartite distinction between computational concepts, practices, and perspectives as its integrative analytical criterion; and (ii) to update the conception of computational talent by extending its definition from the ‘rule-based’ to the ‘data-driven’ and ‘quantum’ stages, distinguishing talent from mere proficiency at each stage through a triple criterion—quantitative, qualitative, and social. As a conceptual contribution, this essay aims to provoke reflection and discussion within the field, leaving its empirical validation for future research.

## 1. Introduction

Computational Thinking (CT) can be defined as the human ability to solve problems and express ideas by relying on concepts, practices, and perspectives of Computer Science (CS) (Moreno-León et al., 2019). Therefore, CT should be understood as a psychological—mainly cognitive—variable, rather than as a merely technical or instrumental skill. Computer programming is probably the main way in which CT comes alive, since it enables individuals to externalize, test, refine, and communicate computational ideas. Nevertheless, CT should not be reduced to programming. While programming is an instrumental competence, CT refers to the underlying cognitive ability that supports and gives meaning to such competence.

This distinction can be clarified through an analogy. Just as verbal aptitude is not identical to reading and writing, although it is deeply involved in them, CT is not identical to computer programming, although it is commonly manifested through it. Verbal aptitude may be projected into multiple tasks involving semantic reasoning, comprehension, or expression. Similarly, CT may be projected into diverse problem-solving situations that require decomposition, abstraction, sequencing, modeling, or algorithmic representation, even when no formal programming task is directly involved (Brackmann et al., 2017; Román-González et al., 2022).

However, CT is not a fixed or ahistorical reality. Unlike physical or biological variables, such as weight, height, or blood type, psychological variables—such as intelligence, creativity, or CT—are constructs. Constructs do not possess an objective and independent existence in the same sense as physical variables. Rather, they are conceptual tools that must be defined, operationalized, and progressively validated by a given scientific and educational community. For this reason, constructs have a history: they emerge, are discussed, are refined, and may eventually reach a certain degree of theoretical and empirical maturity (Román-González et al., 2022).

This point is especially relevant in the case of CT. If CT is defined by reference to the concepts, practices, and perspectives of CS, then its boundaries cannot remain unchanged while CS itself evolves. In other words, CT is a dynamic psychological construct that must evolve in parallel with the development of Computer Science and with the transformation of programming and computing paradigms (Román-González et al., 2025). During the last decade, this evolution has become particularly visible. CT was initially associated with rule-based programming and deterministic logic; more recently, it has expanded toward data-driven approaches linked to Artificial Intelligence (AI) and Machine Learning (ML); and, at an emerging stage, it is beginning to be connected with Quantum Computing (QC) and quantum literacy (Román-González et al., 2025).

A similar argument can be made regarding talent. Talent should not be understood as a fixed, neutral, or merely innate attribute. Rather, talent is also a socially situated construct: what counts as talent in a given historical period depends on the domains that a society values, the forms of performance it recognizes as exceptional, and the educational opportunities it provides for such potential to be cultivated. Contemporary models of giftedness and talent development have progressively moved beyond static and exclusively psychometric conceptions, emphasizing instead the interaction between cognitive abilities, domain-specific performance, creativity, motivation, psychosocial variables, educational opportunities, and sociocultural contexts (Robres & Lozano Blasco, 2020).

This distinction is particularly clear in Gagné’s Differentiated Model of Giftedness and Talent (DMGT), where giftedness refers to outstanding natural abilities, whereas talent designates the outstanding mastery of systematically developed abilities, skills, and knowledge in a particular field of human activity (Gagné, 2004). From this perspective, talent is not simply possessed; it is progressively developed through learning, training, and practice, and is mediated by intrapersonal catalysts, environmental catalysts, and chance (Gagné, 2004). Therefore, talent always implies both an initial disposition and a developmental trajectory.

Subotnik, Olszewski-Kubilius, and Worrell’s developmental approach reinforces this idea. Giftedness is defined as the manifestation of performance or production located at the upper end of the distribution in a specific talent domain, even when compared with other high-functioning individuals in that same domain. Moreover, giftedness changes its form across development: in the early stages, potential is the key variable; later, achievement becomes the main indicator; and, in fully developed talents, eminence becomes the basis for the label (Subotnik et al., 2011). Thus, talent is domain-specific, socially valued, developmentally situated, and dependent on the coalescence of biological, pedagogical, psychological, and psychosocial factors.

Consequently, computational talent should be approached as a domain-specific form of talent within the field of Computer Science Education. It cannot be reduced either to general high intellectual ability or to an isolated programming skill. Rather, it should be understood as a progressively developed capacity to perform at an outstanding level in computationally mediated problem-solving and idea-expression tasks. In previous work, computationally talented students—or computational top thinkers—were operationalized as those students able to accelerate from visual block-based programming environments to text-based programming ones (Román-González et al., 2018b). This criterion was theoretically and empirically useful within a rule-based CT framework, where programming progression could be understood as a movement from introductory visual syntax toward more complex textual programming languages.

Nevertheless, if CT has expanded beyond its initial rule-based stage, the definition of computational talent should be revisited accordingly. A definition limited to the transition from block-based to text-based programming may no longer be sufficient to capture the full range of high computational ability in the current and near-future landscape of CS education. The emergence of AI, ML, Generative AI, and QC suggests that computational talent may now involve not only deductive and algorithmic reasoning, but also inductive and probabilistic reasoning, critical interaction with data-driven systems, iterative and reflective prompting, tolerance of uncertainty, and the capacity to engage with non-binary or quantum ways of representing information.

Following this rationale, the present theoretical article pursues two main objectives. First, it aims to further discuss how CT has evolved during the last decade in parallel with recent developments in CS, through three broad stages: rule-based CT, data-driven CT, and quantum CT. Second, it aims to extend the definition of computational talent by theorizing how it may be manifested in each of these three stages. In other words, this article asks who the computationally talented subjects—or top computational thinkers—are in each stage of the evolving CT framework.

The analytical architecture used to develop this three-stage extension is anchored in Brennan and Resnick’s (2012) framework, which distinguishes computational concepts, computational practices, and computational perspectives. This framework was chosen, rather than other available characterizations of CT, for two main reasons. First, it offers a stable tripartite structure flexible enough to accommodate successive computing paradigms, which allows the same analytical grid to be projected consistently onto rule-based, data-driven, and quantum CT without forcing each stage into a different mode of reasoning. Second, and more fundamentally, it adopts a competency-based orientation—articulating a conceptual dimension (CT concepts), a procedural dimension (CT practices), and an attitudinal-axiological dimension (CT perspectives)—that converges with the orientation recommended by international bodies such as UNESCO for framing educational competencies more broadly. Extending this same tripartite structure across the three stages therefore provides the explicit integrative criterion for the framework developed in Section 2.

The three-stage CT framework can be read from complementary perspectives: as historical phases in the evolution of Computer Science Education, as developmental stages that may guide increasingly complex learning trajectories, as overlapping modes of reasoning that coexist in authentic computational practices, and as a conceptual taxonomy for organizing different forms of CT and computational talent. In this sense, rule-based, data-driven, and quantum CT should be understood as analytically distinguishable but educationally interconnected forms of computational reasoning.

It should be noted, from the outset, that this article is conceived as a theoretical essay. Its aim is to articulate a coherent conceptual framework capable of provoking reflection and discussion within the Computer Science Education and gifted-education communities, and of suggesting new lines of empirical inquiry, rather than to report new empirical findings. Accordingly, the arguments advanced throughout the manuscript combine established empirical evidence—particularly robust for Stage #1, more limited for Stage #2, and still emergent for Stage #3—with theoretical interpretation and extrapolation across stages. The true educational and psychometric scope of this extended framework, and of the definition of computational talent proposed here, can only be determined through subsequent empirical and classroom-based validation, a task that this article leaves explicitly open for future research.

## 2. The Three-Stage Computational Thinking

### 2.1. Definition of Stage #1: Rule-Based Computational Thinking

The first stage of the proposed framework can be defined as rule-based computational thinking. This stage refers to the original and most consolidated educational configuration of CT, closely related to rule-based programming, procedural programming, and the classical paradigm of binary computing. In this initial phase, CT is mainly conceived as a deductive thinking exercise, in which learners formulate a solution by explicitly declaring the rules, steps, conditions, and commands that the machine must execute. Thus, the computational solution is not inferred from data nor generated through probabilistic learning; rather, it is previously designed, formalized, and translated into an executable sequence of instructions.

This interpretation is coherent with Wing’s (2006) seminal conceptualization of computational thinking as a way of solving problems, designing systems, and drawing on concepts fundamental to Computer Science. Nevertheless, in this first stage, such problem-solving is mainly expressed through a rule-based, procedural, and imperative logic. The learner’s task is to make the procedure sufficiently explicit, coherent, and complete for the machine to execute it univocally. Therefore, Stage #1 represents the most classical educational form of CT: a form in which computational ideas become visible through sequences, algorithms, control structures, and predefined instructions.

From a cognitive perspective, rule-based CT follows a predominantly top-down, deductive, and imperative logic. The reasoning process begins with a general goal or problem statement and progressively moves toward particular instructions, exceptions, configurations, and parameterizations. First, learners anticipate the desired behavior; then, they decompose it into smaller operations; finally, they encode each operation so that it can be carried out by the computer. This structure mirrors rule-based programming, where the relevant rules and steps must be declared in advance for the machine to execute them.

This first stage is also grounded in a deterministic philosophical paradigm. Under the same initial conditions, the same series of orders and commands is expected to produce the same result. Computation is therefore understood as the univocal execution of explicitly defined procedures. Consequently, the intelligence of the process lies primarily in the prior formalization of the solution: the learner must specify what the machine has to do, in what order, under which conditions, and with which possible variations. This is what distinguishes rule-based CT from later stages of computational thinking, in which computation is increasingly shaped by data-driven inference, probabilistic reasoning, or non-classical models of computation.

Educationally, this early framework is inseparable from the expansion of visual block-based programming languages, such as Scratch^1^, App Inventor^2^, and Snap!^3^. These environments reduced the syntactic barriers traditionally associated with programming and allowed learners to focus more directly on the underlying thinking processes. Brennan and Resnick (2012) describe Scratch as a programming environment in which young people create interactive stories, games, animations, and simulations by assembling programming blocks. In this sense, block-based programming made procedural structures visible, manipulable, and pedagogically accessible.

This helps explain why the original coining of CT became so successful in educational contexts. Román-González et al. (2019) argue that CT has functioned as an umbrella term that helped place Computer Science Education beyond computer programming. More specifically, CT shifted the educational focus from programming syntax to the mental processes that support programming, lowered the entry barriers to Computer Science, contributed to the CS4all movement, and strengthened the metaphor of programming to learn rather than merely learning to program (Román-González et al., 2019). Therefore, in Stage #1, programming remains central, but its educational value lies in the cognitive structures it makes visible: sequencing, decomposition, abstraction, debugging, modularization, and algorithmic control.

This educational interpretation is also consistent with Kong et al. (2019) overview of computational thinking education. These authors present CT education as a field particularly relevant for K-12 schooling and organized around several interconnected areas: tool development, programming education, student competency and assessment, STEM and non-formal learning, teacher and mentor development, and educational policy. From this perspective, Stage #1 can be understood as the first curricular and pedagogical consolidation of CT: the moment in which computational thinking becomes teachable through accessible programming tools, structured learning experiences, and assessment-oriented frameworks.

Accordingly, Stage #1 can be clearly identified and exemplified through the Brennan and Resnick (2012) framework, which was developed in the context of Scratch programming. Their model organizes computational thinking into three interrelated dimensions: computational concepts, computational practices, and computational perspectives. The seven computational concepts are sequences, loops, events, parallelism, conditionals, operators, and data; these correspond closely to the conceptual structures of rule-based programming. The four sets of computational practices are being incremental and iterative, testing and debugging, reusing and remixing, and abstracting and modularizing; these describe the ways in which learners engage with and refine their computational designs. Finally, the three computational perspectives are expressing, connecting, and questioning, which refer to the ways in which learners come to understand themselves, others, and the technological world through computational creation (Brennan & Resnick, 2012).

In sum, rule-based CT constitutes the foundational stage of computational thinking in educational contexts. It is characterized by procedural programming, deductive and imperative reasoning, explicit rule declaration, deterministic execution, and visual block-based environments. Its main contribution lies in making computational structures visible and accessible, allowing learners to transform ideas into executable procedures. However, its scope remains bounded by the logic of predefined rules: the machine executes what has been previously specified. This limitation prepares the conceptual transition toward later stages of CT, in which computation no longer depends exclusively on explicit procedures, but progressively incorporates data-driven learning, probabilistic inference, and non-classical forms of computational reasoning.

### 2.2. Definition of Stage #2: Data-Driven Computational Thinking

The second stage of the proposed framework can be defined as data-driven computational thinking. This stage refers to the expansion of CT in response to one of the most disruptive developments in contemporary Computer Science: Artificial Intelligence (AI), and more specifically, Machine Learning (ML). Unlike rule-based CT, where the computational solution is explicitly designed in advance through rules, commands, conditions, and control structures, data-driven CT is grounded in the construction, training, validation, and evaluation of computational models from data. Thus, the learner’s task is no longer limited to declaring what the machine must execute; rather, it also involves understanding how a computational system can infer patterns, classify cases, make predictions, or generate outputs from examples.

This interpretation extends, rather than replaces, the classical educational configuration of CT described in Stage #1. If rule-based CT is mainly expressed through procedural programming and deterministic execution, data-driven CT introduces a complementary form of computational reasoning based on data, probability, and model performance. In this sense, the transition from Stage #1 to Stage #2 marks a conceptual movement from programming explicit procedures to building, interpreting, and evaluating data-driven models. The machine is no longer only an executor of predefined instructions; it becomes a system capable of identifying regularities in data and producing probabilistic outputs.

From a cognitive perspective, data-driven CT follows a predominantly bottom-up, inductive, and probabilistic logic. Whereas rule-based CT begins with a predefined procedure and moves toward its execution, data-driven CT often begins with examples, datasets, and observed patterns, from which the system generates a model. Rodríguez-García et al. (2020) explain that ML becomes especially relevant when a problem cannot be precisely solved through a finite set of explicit computations, but there are sufficient data from which useful patterns can be inferred. Consequently, the computational process becomes less dependent on deterministic correctness and more closely related to approximation, inference, uncertainty, bias, and model validation.

This shift has been interpreted by other authors as a substantial reconfiguration of CT itself. Tedre (2022) refers to this movement as Computational Thinking 2.0, arguing that ML challenges several assumptions of classical CT, including control structures, problem-solving workflows, correctness, and notional machines. Similarly, Grover (2024) highlights the need to provide guidance for teaching AI to K-12 learners, drawing on lessons from early AI education and Computer Science education efforts. More recently, Zhong et al. (2026) have also argued that K-12 AI education should integrate CT as an organizing principle, moving beyond tool demonstration and code production toward data- and model-centered problem-solving, responsible human–AI collaboration, and ethics-aware assessment. Therefore, Stage #2 should be understood as a new educational configuration of CT in which learners reason with algorithms, data, models, probabilities, and socio-technical consequences.

Accordingly, Stage #2 should be understood as an extension of the classical CT framework described in Stage #1. The tripartite structure proposed by Brennan and Resnick (2012)—computational concepts, computational practices, and computational perspectives—remains useful because it offers a stable architecture for tracing how CT evolves across different computing paradigms. However, the emergence of AI and ML introduces forms of computational reasoning that cannot be fully explained from a strictly rule-based perspective. Therefore, this second stage does not abandon the classical framework, but expands it to include the specific concepts, practices, and perspectives involved in data-driven model construction, validation, and evaluation.

This extension has already been formulated in the context of AI and ML education. Rodríguez-García et al. (2020), drawing on previous work by Brummelen et al. (2019), identify three new computational concepts for this stage: classification, prediction, and generation. These concepts refer to the different ways in which AI/ML models produce or present their outputs. In parallel, training, validating, and testing emerge as computational practices, since they describe the tasks through which ML models are built, adjusted, and examined. Finally, evaluating becomes a computational perspective, insofar as learners must question whether the final product that integrates the AI/ML model adequately fulfils its intended purpose. This extension preserves the structure of the original CT framework while adapting it to the distinctive logic of AI-based computation.

Educationally, this second stage is inseparable from the development of tools that make AI and ML accessible to young learners. Just as visual block-based programming languages such as Scratch, App Inventor, and Snap! were crucial for the consolidation of Stage #1, platforms such as LearningML^4^ and Machine Learning for Kids^5^ have contributed to bringing Stage #2 into pre-university classrooms. Rodríguez-García et al. (2020) present LearningML as a free, open-source, web-based platform designed to introduce supervised ML through practical AI projects. The tool includes an ML platform, where learners build models from data they collect and label, and a programming platform, where they can develop creative applications using those models. Its design follows the principles of “low floor, high ceiling, and wide walls”, seeking to make ML accessible while allowing increasingly complex projects.

Previous and subsequent studies reinforce this educational trajectory. Rodríguez-García et al. (2019) explored the use of Machine Learning for Kids to develop CT at school, while Rodríguez-García et al. (2020) later evaluated an online intervention designed to teach AI with LearningML to students aged 10 to 16. Taken together, these works suggest that practical ML projects can help learners engage with AI not as a distant or opaque technology, but as a computational process that can be explored, tested, questioned, and improved. Therefore, the educational value of Stage #2 lies not merely in introducing students to AI tools, but in enabling them to understand the logic by which data-driven systems are constructed and evaluated.

However, data-driven CT is not limited to the construction of ML models. A second and more recent facet of Stage #2 is related to Generative Artificial Intelligence (GenAI). GenAI can be understood as a type of AI capable of generating text, images, videos, code, or other forms of data, often in response to prompts. Its rapid expansion has transformed educational, social, and cultural practices, especially since the widespread adoption of systems based on Large Language Models (LLMs). García-Peñalvo et al. (2024) describe this development as a new educational reality that requires careful pedagogical reflection, while Yusuf et al. (2024a) analyse GenAI as both a challenge and an opportunity for the future of higher education. These contributions reinforce the idea that GenAI is not a marginal technological novelty, but a major educational transformation with implications for teaching, learning, academic integrity, and the responsible use of AI.

The emergence of GenAI raises a central question for the evolution of CT: can prompting, or prompt engineering, be considered a new computational thinking practice? At first glance, this possibility requires caution. At the heart of CT lies the premise that learners should maintain an active relationship with digital technologies: they should not merely consume computational outputs, but understand, shape, question, and transform them. In this sense, GenAI entails an evident educational risk: learners may produce superficial prompts, accept AI-generated responses uncritically, and adopt a passive attitude toward systems that may contain errors, hallucinations, or bias. Therefore, prompting should not automatically be equated with CT.

Nevertheless, prompting can be interpreted as a CT practice only when it is carried out in an active, constructive, iterative, and critical manner. In this sense, prompting qualifies as CT when learners formulate a problem, decompose it into constraints or subgoals, evaluate the adequacy and limitations of the AI output, detect errors or hallucinations, refine the prompt on the basis of feedback, and redesign their strategy through successive iterations. Repenning and Grabowski (2023) propose the term “proompting” to describe the iterative process of writing or modifying prompts addressed to AI systems such as ChatGPT or Midjourney. From this perspective, proompting is not a simple request for an answer, but a cycle of problem formulation, prompt expression, output analysis, and refinement. It therefore preserves one of the essential structures of CT: the convergence between human reasoning and computational affordances through iterative problem-solving.

This interpretation is further developed by Hsu (2025), who argues that LLM-based GenAI enables a movement from programming to prompting through natural language programming and prompt engineering. This does not imply that programming disappears, but rather that natural language becomes a new interface for computational expression. By reducing some syntactic and semantic barriers associated with traditional programming languages, LLM-based GenAI may allow learners to focus more directly on problem formulation, decomposition, abstraction, evaluation, and iterative refinement. In this way, prompting may enrich, rather than replace, previous constructionist approaches to CT education.

Hsu (2025) also proposes a constructionist prompting framework organized around five principles: meaningful prompting, metacognitive prompt–response analysis, iterative prompting improvement, social prompting, and learner-directed prompting. These principles help distinguish educationally valuable prompting from merely instrumental or passive interaction with AI. Prompts can become artefacts for thinking: external representations of learners’ ideas that can be analysed, discussed, improved, and connected to meaningful learning tasks. Moreover, Hsu maps core CT elements onto prompt engineering processes: abstraction is linked to concise and explicit prompts; algorithmic thinking to logical and step-by-step prompt structures; debugging to identifying and correcting errors in AI responses; decomposition to breaking complex problems into manageable prompts; generalisation to transferring prior knowledge to new prompting situations; and iteration to refining prompts through feedback.

Finally, the relationship between Stage #1 and Stage #2 should not be understood as a simple replacement of programming by AI. Rather, both stages can intertwine and mutually enrich one another. In this sense, GenAI can operate as a data-driven layer that supports learners’ engagement with rule-based programming tasks. Kim et al. (2024), for example, present App Planner, a generative AI tool designed to support K-12 students in mobile app development. Through guided conversations, the system helps learners articulate problems, brainstorm ideas, receive feedback, and consider the human implications of their designs. Thus, GenAI does not eliminate programming; instead, it can scaffold the planning, conceptualization, and ethical reflection that precede or accompany rule-based app development.

A similar interaction between both stages can be observed in CoRemix. Chen et al. (2025) present a generative AI community-support system designed to help novice programmers understand and remix user-generated projects in the Scratch online community. The system uses visual flowcharts to clarify project logic and combines a prompting pipeline with visual-textual scaffolding, static project analysis, and retrieval-augmented generation (RAG), allowing the AI system to retrieve relevant project information before producing explanations or guidance. According to the authors, CoRemix helped learners understand complex projects, identify event sequencing and relationships across sprites and scripts, strengthen computing-concept skills, and develop better remix strategies. This example is particularly relevant because it shows how a Stage #2 technology—GenAI supported by project analysis and retrieval-based generation—can enhance learners’ access to Stage #1 structures, such as events, scripts, sequencing, project logic, and remixing.

Therefore, the boundary between Stage #1 and Stage #2 should not be conceived as rigid. Data-driven CT can function as a scaffold for deepening rule-based CT: it can help learners plan, explain, debug, remix, and critically improve programming artefacts through AI-mediated support. In this way, Stage #2 should not be interpreted as a departure from programming, but as an expansion of the computational ecosystem in which programming remains relevant while being increasingly supported by data-driven and generative technologies.

Taken together, data-driven CT constitutes the second stage in the evolution of computational thinking. It is characterized by AI and ML, inductive and probabilistic reasoning, data-based model construction, training and validation processes, evaluation of model performance, and iterative interaction with generative systems. Its main contribution lies in expanding CT beyond the logic of explicit rules while preserving the learner’s active role in computational problem-solving. In Stage #1, the machine executes what has been previously specified; in Stage #2, the machine also infers, predicts, classifies, generates, and responds from data. This transition opens a new educational horizon: students must learn to design programmable procedures while also developing the capacity to reason critically with data-driven and generative systems.

### 2.3. Definition of Stage #3: Quantum Computational Thinking

Both Stage #1, rule-based CT, and Stage #2, data-driven CT, belong to the classical computing paradigm. In both cases, information is encoded and processed through binary units, namely bits that take the value of either 0 or 1. The development of quantum computing introduces a new computational paradigm in which information is represented through qubits, understood as quantum states that can be expressed as linear combinations of basis states. Whereas the bit represents information through mutually exclusive states, the qubit introduces a different informational structure: before measurement, its state can be represented as a combination of basis states, and its computational value depends on how this state is transformed, interfered with, and finally measured. This change does not merely concern the speed or power of future machines; it modifies the way information is represented, transformed, measured, and interpreted (Xenakis et al., 2023; Yusuf et al., 2025).

This shift has direct implications for the evolution of computational thinking. In rule-based contexts, learners reason through explicit instructions, sequences, loops, events, conditions, and predictable transformations. In data-driven contexts, they reason through training data, model construction, prediction, validation, and error evaluation. In quantum contexts, computational reasoning must incorporate concepts such as superposition, entanglement, interference, coherence, quantum gates, circuits, and measurement. This shift is not primarily a matter of adding new content to existing curricula, but of overcoming classical intuitions about state, determinism, and debugging, and developing forms of reasoning grounded in probability, measurement, and interference (Farooq & Upadhyay, 2025). Therefore, Stage #3 can be conceptualized as Quantum CT, that is, the extension of computational thinking into the paradigm of quantum information science and quantum computing.

This proposal does not imply that K-12 students should master the mathematical formalism of quantum mechanics. Rather, it suggests that educational systems may progressively introduce the conceptual, practical, and epistemic structures that make quantum computation intelligible. This distinction is important because quantum literacy has been defined not as simple awareness of quantum technologies, but as the acquisition of a minimal body of fundamental knowledge that enables individuals to understand possible uses of quantum computation across different domains and to evaluate claims about quantum technologies with scientific caution (Nita et al., 2023).

The educational relevance of this stage is increasingly visible. Quantum technologies are becoming a strategic priority in scientific, industrial, and political agendas, while quantum education remains only partially developed in school contexts. Halfhill et al. (2026) identify a clear gap between national and international quantum strategies and their translation into classroom-level curricula, noting that research on quantum education in primary and secondary settings is still scarce when compared with broader STEM education literature. This gap reinforces the need for educational proposals that are scientifically accurate, pedagogically feasible, and aligned with existing curricular structures (Halfhill et al., 2026).

This educational relevance is also supported by an early systematic review on quantum computing in K-12 education, which examined empirical research in this field. The review shows that the introduction of quantum computing in school education has been justified through several converging reasons: its strategic national importance, its interdisciplinary relevance, the need for early exposure and familiarity, diversity and inclusion, career preparation and workforce development, and the future potential of quantum technologies. These findings reinforce the idea that Quantum CT should not be understood as an isolated technological novelty, but as part of a broader educational response to an emerging scientific and computational paradigm (Yusuf et al., 2024b).

Some authors have therefore argued that quantum education should be introduced gradually and accessibly, without necessarily creating a standalone subject in the early stages. Garcés et al. (2025) propose a modular integration of quantum computing into mathematics, physics, and computer science, using existing curricular entry points such as probability, linear algebra, complex numbers, programming, data representation, physical systems, and computational models. Similarly, Foti et al. (2021) emphasize that quantum literacy requires pedagogical mediation, since quantum phenomena are not easily grounded in everyday experience and their mathematical language often exceeds the background of non-specialist learners.

Building on Brennan and Resnick’s (2012) distinction between computational concepts, practices, and perspectives, Quantum CT can be organized around three analogous dimensions: quantum CT concepts, quantum CT practices, and quantum CT perspectives. In this stage, the computing paradigm is non-binary, the programming mode is quantum programming, the logic involved is non-classical, and the philosophical orientation can be described, with caution, as holistic, insofar as quantum systems require reasoning about states, relations, measurement, and uncertainty as interdependent aspects of the same phenomenon.

The first dimension, quantum CT concepts, refers to the fundamental ideas that allow learners to understand how quantum computation differs from classical computation. The selection of these concepts is consistent with the current empirical landscape of K-12 quantum computing education. An early systematic review identified fourteen quantum concepts addressed in K-12 studies, grouped into foundational concepts—qubit, superposition, entanglement, measurement, quantum state, and interference—and computational concepts—quantum gates, circuits, teleportation, probability, reversibility, algorithms, encryption, and cryptography. The same review found that superposition, entanglement, and measurement were the most frequently studied concepts, whereas more advanced computational notions, such as cryptography and encryption, tended to appear mainly at upper-secondary levels (Yusuf et al., 2024b).

Following Xenakis et al. (2023), seven concepts are particularly relevant for this stage: qubits, superposition, quantum advantage, entanglement, teleportation, quantum gates, and tunnelling. A qubit can be understood as the quantum counterpart of the classical bit, although it is not restricted to the binary alternatives of 0 or 1; rather, it can be represented as a linear combination of basis states. Superposition refers to the possibility that a quantum system may be described through more than one potential state before measurement. Quantum advantage points to those cases in which a quantum device can solve a problem that would be infeasible for classical computers within a reasonable time. Entanglement describes a form of correlation between quantum systems that cannot be fully explained through classical independence; more precisely, the measurement of one part of an entangled system constrains the possible state of the other, even when the systems are spatially separated. Teleportation refers to the transfer of quantum information from one system to another through a shared entangled state, together with the classical communication needed to complete the protocol. Quantum gates operate as transformations over quantum states and, when organized into circuits, make quantum computation procedurally expressible. Finally, tunnelling refers to the possibility that a quantum system crosses a potential barrier that would not be passable from a classical perspective. Together, these concepts provide the conceptual grammar through which learners can begin to reason about quantum information, quantum operations, and quantum algorithms (Xenakis et al., 2023).

Interference should also be included as a central concept in this stage, since quantum algorithms depend on the possibility of amplifying desirable probability amplitudes and reducing or cancelling others before measurement. Without this idea, quantum computation may be mistakenly reduced to a simplistic account of “being in several states at once”, when its computational power is better understood as the deliberate transformation of probability amplitudes through quantum operations (Nita et al., 2023).

Recent work on quantum literacy assessment expands this conceptual basis by including additional notions such as reversibility and algorithms, which are especially relevant for K-12 education. Reversibility helps learners understand that many quantum operations preserve information and can, in principle, be undone, whereas quantum algorithms allow students to connect quantum principles with structured procedures for solving computational problems (Yusuf et al., 2025).

The second dimension, quantum CT practices, concerns the forms of activity through which learners engage with quantum systems. These practices include modelling quantum states, predicting probabilistic outcomes, designing and interpreting circuits, running simulations, comparing expected and observed results, reasoning with multiple representations, and testing computational procedures whose results cannot always be understood from a single execution. In this respect, quantum programming introduces a distinctive challenge: the state of a system cannot always be directly inspected without altering it, and the meaning of an output often emerges through repeated measurements and probability distributions (Nita et al., 2023; Xenakis et al., 2023). Farooq and Upadhyay (2025) characterize the cognitive practices this transition demands: experimentation, reflection, prediction, measurement, and debugging oriented toward moving from classical intuitions to quantum models, together with the design and interpretation of quantum circuits.

Within this stage, two practices deserve particular attention: seeking coherence and intentionally collapsing. Seeking coherence refers to the effort to preserve the quantum conditions that allow superposition, entanglement, and interference to remain computationally meaningful. Intentionally collapsing refers to the design and interpretation of measurement processes through which a quantum state is converted into an observable outcome. These practices should not be understood as mechanical classroom actions, but as cognitive and computational orientations that help learners reason about when quantum possibilities are preserved, transformed, or resolved through measurement.

The third dimension, quantum CT perspectives, refers to the epistemic attitudes that emerge when learners reason computationally within the quantum paradigm. These perspectives include assuming uncertainty, understanding the role of measurement, recognizing the limits of direct observation, and interpreting information as physically instantiated rather than merely symbolic. Moreover, the perspective of unity within diversity may be understood as the ability to recognize that apparently separate quantum systems can form relational structures whose behavior cannot be fully explained by analyzing each component in isolation. In this limited but meaningful sense, Stage #3 introduces not only new concepts and practices, but also a new way of thinking about the relation between information, reality, and knowledge.

The development of these concepts, practices, and perspectives requires carefully designed educational mediation. Since one of the main barriers to quantum learning is the density of its mathematical formalism, visual, game-based, puzzle-based, unplugged, and block-based approaches may offer valuable entry points (Escanez-Exposito et al., 2025). Platforms and tools such as Quarks Interactive^6^, Qubit by Qubit^7^, QPlayLearn^8^, QScratch^9^, and other quantum games seek to make quantum ideas more accessible while preserving scientific accuracy. Foti et al. (2021), for example, present QPlayLearn as a multilevel platform that combines playful, experiential, and formal approaches, allowing learners to move gradually from intuition to more refined forms of understanding.

This is consistent with the available synthesis of K-12 quantum computing education, which shows that games are the most frequently used tools, followed by simulators, unplugged tools, reading materials, programming tools, visuals, practice sheets, and hardware tools. However, this evidence also suggests that these tools must be carefully scaffolded, since highly interactive or game-based environments may increase engagement while also imposing considerable cognitive demands on learners (Yusuf et al., 2025).

Nita et al. (2023) also emphasize the role of visual and puzzle-based tools in reducing the mathematical barrier to entry. Their proposal is especially relevant because it does not treat visualization as a superficial simplification, but as a way of supporting rigorous intuition about quantum processes. In this line, visual models may help non-specialist learners approach concepts such as interference, state transformation, and quantum computation without requiring immediate mastery of advanced symbolic notation (Nita et al., 2023).

Empirical and design-based studies further suggest that quantum concepts can be connected to computational thinking through problem-based and game-based learning. Xenakis et al. (2023) propose quantum serious games in which students use Python and quantum concepts to design and test strategic games such as quantum tic-tac-toe and quantum naval battleship. In these contexts, students engage with superposition, entanglement, gates, measurement, and quantum information while practicing abstraction, decomposition, modelling, hypothesis testing, debugging, and evaluation (Xenakis et al., 2023).

The recent development of the Quantum Literacy Test also strengthens the theoretical plausibility of Stage #3. Yusuf et al. (2025) designed and validated this instrument to assess upper-secondary students’ understanding of quantum concepts, practices, and perspectives. Their findings showed strong internal consistency and substantial concurrent validity with a Computational Thinking Test. At the same time, the fact that computational thinking and spatial ability explained less than 40% of the variance in quantum literacy suggests that this domain may involve specific forms of reasoning that cannot be fully reduced to classical CT or to general cognitive ability.

Therefore, Stage #3 expands the CT framework by incorporating a new computational paradigm and a new epistemic horizon. Quantum CT refers to the progressive development of computational reasoning within a paradigm in which information is represented through quantum states, transformed by quantum operations, affected by measurement, and interpreted through probabilistic outcomes. In this sense, Stage #3 broadens the CT framework by incorporating the conceptual and epistemic demands of quantum computation: superposition, entanglement, coherence, interference, gates, circuits, algorithms, and measurement. Thus, Quantum CT represents a further evolution of computational thinking: from deterministic rule-following, to probabilistic model-building, and finally to quantum reasoning about information, uncertainty, coherence, and measurement (see Table 1).

## 3. Computational Talent

After outlining the three stages of computational thinking, this section examines how computational talent may be identified within each of them. The aim is to specify the forms of advanced, observable, and educationally meaningful performance that distinguish computational talent from general computational proficiency.

Before characterizing computational talent within each stage, it is useful to clarify the general criteria through which this distinction can be approached. Drawing on the classical distinction established in the gifted-education literature (Tourón, 2024), computational talent may be differentiated from mere computational proficiency according to three complementary criteria: a quantitative criterion, a qualitative criterion, and a social criterion.

From a quantitative standpoint, computational talent is reflected in the capacity to reach the learning milestones associated with each stage with greater speed, earlier precocity, and less instructional support than would typically be expected. Talented learners do not merely attain the same outcomes as proficient learners; they tend to attain them earlier and with greater autonomy.

From a qualitative standpoint, computational talent involves a depth, complexity, and sophistication of learning and performance within each stage that goes beyond general proficiency. In this case, the distinction does not primarily concern pace, but rather the representational, conceptual, and cognitive reach of the learner’s performance.

A third social criterion complements the previous two. Computational talent may also be distinguished by the learner’s capacity to communicate, share, and make meaningful use of their productions with others, thereby socializing their achievements within relevant communities of practice. In this sense, computational talent is not only expressed in what learners are able to understand or produce individually, but also in how they are able to articulate, explain, and transfer their work in socially meaningful contexts.

This triple criterion connects directly with classical models of educational intervention for gifted students (Tourón, 2024): acceleration, which is primarily associated with the quantitative criterion; enrichment, which corresponds to the qualitative criterion; and grouping, which relates to the social criterion.

These three criteria can now be applied to computational talent within each of the three stages of the proposed model. To make this characterization more concrete, illustrative examples will be provided for each stage, situated around Grade 8, approximately 14 years of age. These examples illustrate possible manifestations of computational talent and show how advanced performance becomes visible in educationally meaningful ways at a specific developmental and curricular level.

### 3.1. Computationally Talented Students in Stage #1

Within Stage #1, computationally talented students, or computational top thinkers, may be initially conceptualized as those learners who show an outstanding command of the CT concepts, CT practices, and CT perspectives that define this rule-based stage. It refers to the capacity to use sequences, loops, events, parallelism, conditionals, operators, and data in increasingly efficient, flexible, and meaningful ways; to engage fluently in incremental design, testing, debugging, reusing, remixing, abstracting, and modularizing; and to adopt computational perspectives related to expressing, connecting, and questioning (Brennan & Resnick, 2012).

Thus, in the context of rule-based CT, computational talent can be described as an advanced capacity to transform ideas into explicit and executable procedures. These students anticipate the structure of the solution, organize the sequence of actions, identify errors, refine their code, and move progressively toward clearer, more elegant, and more modular solutions. In other words, they are especially proficient in the deductive, top-down, and imperative reasoning that characterizes Stage #1.

A second way to identify computational top thinkers in this stage is through accelerated progression from visual block-based programming to text-based programming. Prior empirical work operationalized computational talent in middle school through students’ spontaneous acceleration from a block-based environment, such as Code.org, to a text-based environment, such as Khan Academy’s JavaScript course (Román-González et al., 2018a). This study showed that computationally talented students could be detected before formal coding instruction and suggested that these learners may accelerate one or two years in relation to Computer Science Education standards. However, the same work also warned that this criterion may be gender biased when acceleration depends on self-selection or explicit student request (Román-González et al., 2018a).

This acceleration reflects a deeper cognitive movement that exceeds a merely technical transition from one interface to another: from manipulating visible blocks toward managing more abstract symbolic structures. In Stage #1, therefore, computationally talented students are those who can move earlier and more effectively from concrete visual procedures to more formalized, textual, and syntactically demanding representations of algorithms.

This interpretation is also consistent with a later developmental approach to CT assessment, in which computational thinking is described as a progression through several cognitive subprocesses: decomposition, pattern recognition, abstraction, algorithmic design, automation, evaluation, and generalization (Román-González & Pérez-González, 2024). In that framework, CT development is presented as a progressive organization of cognitive subprocesses and algorithmic structures across educational stages. Middle school appears as a particularly relevant transition point: students begin to consolidate abstract thinking, metacognitive regulation, systematic problem-solving, debugging, modularization, and nesting. From this perspective, computationally talented students may be understood as those who progress faster through this developmental sequence and perform, at an earlier age, in ways expected from later stages.

A third and more cognitively demanding aspect concerns abstraction. If abstraction is considered one of the core processes of computational thinking, then computational top thinkers in Stage #1 would be those able to operate at higher levels of abstraction within rule-based programming. This means filtering irrelevant information, identifying structural similarities, mapping the relationships between the main problem and its subproblems, and representing the solution at an appropriate level of generality. Qian and Choi (2023) describe abstraction in CT as a process that involves filtering information, locating similarities, and mapping problem structures, while also moving across multiple layers of abstraction. From this perspective, computational talent involves representational depth as much as speed or performance.

At the upper boundary of Stage #1, this capacity for abstraction may even appear in advanced programming practices such as metaprogramming, where learners begin to treat programs as manipulable objects. Recent work on Snap! metaprogramming describes the possibility of writing programs that generate, read, or modify other programs, thereby allowing students to reason about the structure of procedures themselves rather than only about their execution (Moreno-León et al., 2025). Although metaprogramming remains uncommon in ordinary K-12 rule-based CT, it offers a useful horizon for understanding the most advanced learners within this stage: computational top thinkers write procedures and, further, begin to reason about procedures as objects of thought.

Accordingly, the triple criterion introduced above can help distinguish general rule-based CT proficiency from computational talent in Stage #1. From a quantitative point of view, computational talent is expressed in an earlier and faster transition from visual programming languages, such as Scratch, to textual programming languages, such as Python. From a qualitative point of view, computational talent is expressed in the capacity to think and act in terms of metaprogramming: talented learners conceive and use their own programs as objects that can, in turn, become part of other, more complex and abstract (meta)programs—a leap in abstraction that proficiency alone does not reach. From a social point of view, computational talent is expressed in sharing programs in open repositories, so that they are actually used, remixed, and improved by third parties; talented learners communicate effectively within programmer communities. These three criteria function as complementary and convergent signs of advanced performance within the procedural and deterministic logic of this first stage. Table 2 synthesizes this distinction within the broader three-stage framework of computational talent.

In sum, computationally talented students in Stage #1 may be defined as those learners who show exceptional mastery of rule-based CT. They use CT concepts, practices, and perspectives with fluency; they accelerate from block-based to text-based programming; they operate at increasingly higher levels of abstraction, including incipient metaprogramming; and they share and discuss their productions within programmer communities. Their talent is expressed in designing clearer, more efficient, more modular, and more transferable computational solutions, and in making those solutions genuinely available to others, within the deterministic and procedural logic of this first stage.

### 3.2. Computationally Talented Students in Stage #2

Within Stage #2, computationally talented students, or computational top thinkers, may be conceptualized as those learners who show an outstanding command of the CT concepts, CT practices, and CT perspectives that define data-driven computational thinking. In this stage, talent is expressed through the capacity to reason with data, construct and improve models, interpret probabilistic outputs, and critically evaluate the behaviour and limitations of AI systems—a capacity that exceeds the explicit rule declaration and deterministic procedure design characteristic of Stage #1. Therefore, computational talent in Stage #2 refers to excellence within the specific logic of data-driven computation.

More specifically, computationally talented students in this stage are those who understand and use classification, prediction, and generation in increasingly flexible and meaningful ways. These concepts describe technical outputs of AI/ML models and also require learners to understand what kind of problem is being addressed, what type of data are needed, and what form of output is expected. Thus, a computational top thinker in Stage #2 can distinguish whether a task requires classifying cases, predicting future or unknown values, or generating new content from learned patterns. This conceptual flexibility is central to data-driven CT, since the outputs of AI/ML models depend on data, context, and probability instead of following predefined procedures.

A second criterion concerns mastery of the computational practices specific to this stage: training, validating, and testing. Computationally talented students move beyond the mere use of an AI tool and understand the process through which a model is progressively built and assessed. They can collect and organize relevant data, label examples coherently, train a model, test its performance, identify errors, and decide whether additional or better data are needed. In this sense, their talent is expressed in model-oriented reasoning: they understand that the quality of the output depends on the quality, quantity, balance, and relevance of the data used to construct the model. This is consistent with the ML workflow described by Rodríguez-García et al. (2020), in which learners build datasets, generate models, evaluate their performance with test data, and integrate them into programming environments.

A third and especially decisive criterion concerns evaluating. In Stage #2, computationally talented students critically question apparently successful AI outputs. They ask whether the model performs well, whether its errors are systematic, whether its predictions are reliable, whether the dataset may be biased, and whether the final AI-based product adequately fulfils its intended purpose. This evaluative capacity is essential because ML models may produce biased or incorrect results even when they appear accurate. Hence, learners must treat AI outputs as probabilistic results that require interpretation, validation, and responsible use.

This evaluative dimension becomes even more important in the context of Generative AI. Lubbe et al. (2025) argue that GenAI disrupts traditional assessment hierarchies because it can perform tasks associated with high levels of Bloom’s taxonomy, especially creative production. As a result, the distinctive human contribution is increasingly located in critical evaluation of the AI-generated product, assessing accuracy, credibility, coherence, originality, bias, and ethical adequacy in AI-generated content. In this sense, computational talent in Stage #2 cannot be inferred from the surface quality of the final product alone. When GenAI can generate fluent, sophisticated, and apparently creative outputs, the most relevant evidence of talent lies in the learner’s capacity to evaluate, justify, refine, and take intellectual responsibility for the process.

This emphasis on epistemic vigilance is also coherent with recent discussions in gifted education. Sternberg (2023) conceptualizes giftedness as a socially recognized interaction between person, task, and situation. In GenAI-mediated contexts, this argument becomes particularly relevant: if the task, the tools, and the conditions of performance are transformed by AI, the evidence of advanced ability must be sought in the learner’s capacity to frame the task, make informed decisions, justify the use of AI-generated outputs, and preserve ownership of reasoning throughout the process, since the polished product alone offers little evidence of it.

In this regard, computational talent in Stage #2 may be connected with a broader conception of advanced ability in the GenAI era. Guilbault et al. (2025) emphasize that gifted education should cultivate learner agency and developmental growth, while Preiss and Sternberg (2025) argue that human excellence in the age of GenAI must preserve critical thinking, creativity, wisdom, and moral judgment. Sternberg (2025, 2026) further suggests that the central educational challenge concerns how learners decide what to delegate to AI and what must remain cognitively and ethically their own, beyond the question of whether AI increases or decreases cognitive performance. Therefore, computationally talented students in Stage #2 govern the human–AI relationship with discernment, responsibility, and intellectual autonomy, well beyond simply using AI faster or more efficiently.

Another expression of talent in Stage #2 appears in the learner’s capacity to engage in high-quality prompting (Repenning & Grabowski, 2023). Since this practice has already been defined as an active, constructive, iterative, and critical form of prompting, the relevant question here concerns what distinguishes a computationally talented learner within this kind of interaction. In this regard, computational top thinkers formulate problems with precision, transform vague intentions into well-structured prompts, decompose complex requests into smaller steps, compare alternative outputs, detect missing information or hallucinations, and progressively guide the AI system toward more adequate responses. This connects with Siegle’s (2025) argument that prompt engineering can improve gifted students’ questioning: the intellectual value of GenAI interaction lies in learning to pose more precise, generative, and cognitively demanding questions, well beyond simply receiving answers. Their talent lies in orchestrating a dialogue in which human judgment remains active.

This form of prompting is closely aligned with Hsu’s (2025) constructionist prompting framework, in which prompts function as artefacts for thinking that exceed mere requests for answers. In talented learners, proompting thus becomes an indicator of computational agency: the capacity to think with the system while preserving intellectual ownership of the direction, quality, and meaning of the process.

At a more integrated level, the most advanced learners in Stage #2 can be understood as those who connect data-driven reasoning, critical evaluation, and generative interaction. They recognize that AI systems are built from data, shaped by models, and constrained by training processes, design decisions, and possible biases, and therefore cannot be treated as neutral or infallible. Consequently, they can move across different layers of reasoning: from preparing data to interpreting outputs, from designing prompts to evaluating responses, and from using AI-generated material to justifying their own intellectual decisions. This movement requires a form of computational maturity that exceeds technical proficiency alone.

A further indicator of computational talent in data-driven CT is the ability to navigate fluidly between data-driven and rule-based forms of computational thinking. GenAI can scaffold the planning, explanation, debugging, and remixing of programming artefacts; talented learners are those who deliberately exploit this complementarity, using AI-based technologies to deepen their command of rule-based structures without treating them as substitutes.

This characterization has important implications for the identification of computational talent. In rule-based CT, talent could be observed through fluency in rule-based programming, acceleration from block-based to text-based environments, abstraction, debugging, and modularization. In Stage #2, the evidentiary value of the final product becomes more ambiguous, because AI systems can contribute substantially to the apparent quality of the output. This ambiguity is empirically reinforced by recent performance-based research on AI literacy: across tasks that required participants to solve problems using ChatGPT, human cognitive abilities explained roughly half of the variance in an information-seeking task, a modest share of the variance in a creative-ideation task, and none of the variance in an argumentative-writing task; self-reported AI usage and attitude toward AI, moreover, were unrelated to actual performance in any of the tasks (Rindle et al., 2026). This pattern suggests that a fluent, well-produced output—and even frequent, confident AI use—offers a poor proxy for the underlying human contribution, particularly in tasks where framing, evaluating, and refining leave the only visible trace of talent. Assessment should therefore attend closely to the learner’s process: model-oriented reasoning, purposeful dataset construction, interpretation of model error, evaluation of bias and reliability, iterative prompting or proompting, and intellectual ownership of AI-assisted outputs, evidenced for instance through prompt-history analysis, reflection logs, oral explanations, dataset or prompt justification, and interpretation of errors. This process-oriented assessment is consistent with recent accounts of GenAI-supported CT, which conceptualize prompting as an epistemic and pedagogical practice capable of making computational reasoning visible, beyond its role as a technical skill (Creely, 2026). In other words, the relevant evidence lies less in what the student produces with AI than in how the student frames the problem, documents decisions, evaluates outputs, revises the process, and remains accountable while using it.

The same triple criterion becomes visible within Stage #2. From a quantitative point of view, computational talent learns faster to build reliable machine learning models: using tools such as LearningML, talented learners more quickly learn to train models on balanced, unbiased datasets and to reach a sufficiently trustworthy predictive model, with low rates of false positives and false negatives, whereas proficient learners reach the same outcome but require more time and effort. From a qualitative point of view, computational talent approaches prompting in a manner that is qualitatively distinct from mere proficiency: owing to a superior metacognitive capacity, talented learners engage in prompting that is more focused, planned, self-regulated, conscious, and iterative. An observable indicator of this quality is that talented learners keep an explicit record, in the form of a prompt-history log, revealing their resistance to converging prematurely on the quick solutions offered by generative AI; proficient learners, by contrast, may prompt effectively without displaying this deeper metacognitive quality. From a social point of view, computational talent is expressed in the capacity to integrate a machine learning model into a program or application—built, for instance, with Scratch or Snap!—that can genuinely be used by third parties or real audiences with whom the learner interacts; a talented learner integrates that model into an application that can be downloaded, used, and discussed by others, going beyond building a model that merely classifies waste by image, for example. Table 2 situates these indicators within the broader three-stage framework.

Accordingly, computationally talented students in Stage #2 may be defined as those learners who show exceptional mastery of data-driven CT. More specifically, they understand and use classification, prediction, and generation; they train, validate, and test AI/ML models; they evaluate probabilistic outputs critically; they interpret model performance and possible bias; they engage in active and iterative proompting; and they connect AI-mediated support with rule-based programming practices. In the GenAI era, their talent becomes visible chiefly in the quality of their problem framing, question design, evaluative judgment, epistemic vigilance, and accountable ownership of reasoning, well beyond the mere production of correct, fluent, or impressive outputs. Thus, computational top thinkers in Stage #2 reason critically, flexibly, and responsibly with data-driven and generative systems while preserving the distinctively human dimensions of agency, creativity, wisdom, and ethical responsibility.

### 3.3. Computationally Talented Students in Stage #3

If Stage #3 is defined by the emergence of Quantum CT, computational talent at this level may be provisionally conceptualized as the capacity to reason fluently within the conceptual, practical, and epistemic conditions of quantum computation. In this sense, computationally talented learners in Stage #3 show advanced engagement with the quantum CT concepts, practices, and perspectives that characterize this new computational paradigm.

At the conceptual level, these learners would be expected to understand the basic grammar of quantum information. They can distinguish bits from qubits, interpret superposition as a state representation prior to measurement, recognize entanglement as a non-classical form of correlation, and understand the role of quantum gates, circuits, tunnelling, teleportation, quantum advantage, interference, reversibility, and algorithms in computational processes. Xenakis et al. (2023) identify several of these notions—qubits, superposition, quantum advantage, entanglement, teleportation, gates, and tunnelling—as central concepts for Quantum Computational Thinking, particularly when learners engage with games and activities related to quantum computing and quantum information processing. Recent work on the Quantum Literacy Test expands this conceptual domain by including reversibility and algorithms as relevant notions for K-12 quantum education (Yusuf et al., 2025).

Conceptual understanding is completed by distinctive quantum CT practices. Computationally talented learners in this stage may be able to model quantum states, predict probabilistic outcomes, design and interpret quantum circuits, run simulations, compare expected and observed results, and reason with multiple representations. These practices impose substantial cognitive and spatial demands, since learners must coordinate abstract state representations, transformations, measurement, and probabilistic distributions that often exceed directly observable processes. In this context, spatial visualization becomes an important cognitive support for helping non-specialist students understand multidimensional models of quantum states, circuit transformations, and measurement outcomes (Dündar-Coecke et al., 2023). Quantum programming introduces a specific cognitive demand: the state of a system cannot always be inspected directly without altering it, and the meaning of an output often emerges through repeated measurements and probability distributions.

Nita et al. (2023) show that the learning of quantum concepts is frequently constrained by mathematical formalism and by the absence of familiar analogies, which makes visual and puzzle-based tools especially valuable for developing rigorous intuition. Recent work on metaphors for quantum computational thinking further supports this idea. Beardow and Stappers (2026) argue that metaphors can act as pedagogical bridges between abstract quantum principles and computational practice, provided that they make explicit the computational role, interdependence, and consequences of quantum concepts, going beyond merely familiar images. In this sense, visual and puzzle-based environments may help learners move beyond intuitive approximation towards a more structured form of quantum computational reasoning.

Within these practices, two abilities may be useful as exploratory indicators of advanced quantum CT: seeking coherence and intentionally collapsing. Seeking coherence involves understanding that quantum computation depends on preserving the conditions that allow superposition, entanglement, and interference to remain computationally meaningful. Intentionally collapsing involves designing, interpreting, and evaluating measurement processes through which a quantum state becomes an observable result. These abilities require learners to reason about possibilities before measurement, transformations during computation, and probability distributions after measurement.

Computationally talented learners in this stage also show a distinctive way of approaching uncertainty. The result of a quantum computation, unpredictable in a single execution, remains mathematically structured and computationally meaningful; their reasoning coordinates uncertainty with rigor, treating probabilistic outcomes as part of the computational process itself. This is consistent with the broader definition of quantum literacy proposed by Nita et al. (2023), who describe it as the minimal body of knowledge needed to understand how quantum computation may be used in different domains and to evaluate claims about quantum technologies.

The educational literature suggests that this form of talent may be cultivated through accessible and progressive learning environments. Foti et al. (2021) argue that quantum literacy requires imagination, interactive tools, and carefully designed mediation, since quantum phenomena exceed the grounding that everyday intuition or simple classroom demonstrations can offer. Similarly, Garcés et al. (2025) propose integrating quantum computing into existing mathematics, physics, and computer science curricula through modular pathways, accessible from pre-university levels onward. Therefore, computational talent in Stage #3 may emerge in learners who show a particular capacity to reason visually, experimentally, relationally, and computationally about quantum phenomena.

At the same time, this characterization should be formulated with caution, since Stage #3 is proposed here as a theoretically grounded frontier for future research, still distant from a consolidated pedagogical or diagnostic reality. The empirical literature on K-12 quantum computing education remains at an early stage: the available systematic evidence reports promising outcomes, such as knowledge acquisition, interest, engagement, enjoyment, satisfaction, motivation, confidence, and performance, yet the overall quality of evidence is still moderate, and many studies rely on case reports or short-term interventions. The identification of computationally talented learners in Stage #3 is therefore best understood as a theoretically grounded and educationally plausible proposal, awaiting fuller empirical validation (Yusuf et al., 2024b).

Game-based and problem-based environments offer especially useful contexts for observing this form of talent. Xenakis et al. (2023) show that students who worked with quantum serious games within a problem-based and Quantum Computational Thinking framework outperformed a control group in quantum problem-solving and in CT-related dimensions such as abstraction, decomposition, testing, and data analysis. In these contexts, learners may show possible indicators of advanced quantum CT, such as the ability to formulate hypotheses, test strategies, revise models, interpret probabilistic feedback, and connect quantum rules with computational structures.

The recent validation of the Quantum Literacy Test provides preliminary support. Yusuf et al. (2025) found that quantum literacy correlates substantially with computational thinking, while computational thinking and spatial ability explained less than 40% of the variance in quantum literacy performance. This finding suggests that Stage #3 talent involves a domain-specific form of reasoning: the computationally talented learner in Quantum CT integrates classical CT abilities with quantum-specific concepts, practices, and perspectives.

This connection is especially relevant for intellectually gifted learners, whose advanced cognitive development may be accompanied by existential questions about meaning, purpose, and the full expression of potential. Introducing learners to uncertainty, relationality, measurement, and the limits of direct observation, Quantum CT can foster a form of transcendent thinking: a disposition to inquire into the nature of knowledge, reality, and meaning, beyond instrumental problem-solving. In this sense, the transcendent dimension of quantum CT connects computational education with deeper forms of intellectual and existential development (Rodríguez-Fernández & Sternberg, 2024).

Within Stage #3, the same triple criterion offers a tentative, exploratory way of describing computational talent, in keeping with the caution called for by the still-emerging state of K-12 quantum computing education (as shown in Table 2). From a quantitative point of view, and analogously to acceleration in mathematics, a computationally talented learner in middle school may already be capable of operating with quantum logic gates, which require matrix operators ordinarily associated with high-school mathematics; proficient learners will also become able to operate these gates, but later, generally toward the end of high school. This form of talent can be observed in the growing number of quantum serious games and puzzle-based environments, where students who resolve high-school-level quantum challenges already in middle school provide a plausible indicator of talent, consistent with the problem-based Quantum Computational Thinking evidence reported by Xenakis et al. (2023) and with recent game-based approaches to quantum learning (Moss & Mehta, 2026; Bondani et al., 2026). From a qualitative point of view, computational talent is expressed in at least three distinguishable ways. First, owing to a greater capacity for abstraction and metacognition, talented learners can consciously decide in which situations or for which problems a quantum approach is preferable to a classical, Newtonian one, deliberately shifting between a quantum mindset and a classical mindset according to the demands of the task. Second, while proficient learners at middle-school level typically approach quantum concepts through metaphors and analogies (Beardow & Stappers, 2026), talented learners can access more abstract levels of representation, such as quantum picturalism (Dündar-Coecke et al., 2026), or even venture into symbolic algebraic representation. Third, talented learners generally handle the counterintuitive paradoxes of quantum mechanics with greater ease than merely proficient learners.

From a social point of view, computational talent is expressed in the capacity to communicate quantum ideas to others and, in particular, to extrapolate core quantum notions—self-organization, holism, and entanglement—to the leadership of groups and the management of organizations. This extrapolation is consistent with Zohar’s proposals of Quantum Management (Zohar, 2022b) and Quantum Leadership (Zohar, 2022a), which replace the mechanistic, top-down logic inherited from Newtonian physics with an organic model in which organizations behave as self-organizing, interconnected living systems, and in which the leader’s role is defined by qualities such as self-awareness, holistic vision, and adaptive spontaneity.

In sum, computational talent in Stage #3 may be tentatively described as quantum computational fluency: the ability to reason flexibly with qubits, superposition, entanglement, interference, coherence, gates, circuits, measurement, and probabilistic outcomes; to interpret simple quantum circuits and compare expected and observed results; and to evaluate claims about quantum technologies with scientific caution. This fluency spans the three dimensions introduced above—quantum CT concepts, practices, and perspectives—and its talented expression lies in moving across them with flexibility, rigor, and creativity. Given the still-emerging state of K-12 quantum education, these indicators remain a theoretically grounded proposal, awaiting further empirical validation. In this sense, Quantum CT talent constitutes a form of epistemic maturity: the capacity to think computationally in a domain where information, probability, physical reality, and measurement are inseparably connected, engaging the transcendent dimension of thought noted above.

Taken together, the preceding characterization of computational talent across Stages #1 to #3 can be synthesized into a single, integrative framework. Table 2 summarizes, for each stage, the general profile that identifies computational top thinkers, together with the way in which the quantitative, qualitative, and social criteria become manifest, alongside their correspondence with the classical models of educational intervention—acceleration, enrichment, and grouping (Tourón, 2024). This synthesis functions as a heuristic map, capable of guiding both the identification of computational talent and the design of differentiated educational responses across the successive stages of computational thinking.

**Table 2:** Computational Talent across three stages.

Stage	Criterion	Description
**Stage #1** **Rule-based CT**	General profile	Exceptional mastery of rule-based CT; efficient and modular procedural solutions; early transition from block-based to text-based programming; faster CT developmental progression; higher abstraction in procedural programming.
	Quantitative	Earlier and faster transition from block-based to text-based programming (e.g., Scratch → Python).
	Qualitative	Metaprogramming: treating one’s own programs as objects that can form part of other, more complex (meta) programs.
	Social	Sharing code in open repositories, used and remixed by third parties; effective communication in programmer communities.
**Stage #2** **Data-driven CT**	General profile	Exceptional mastery of data-driven CT; flexible use of classification, prediction and generation; ability to train, validate and test AI/ML models; critical evaluation of bias, reliability and probabilistic outputs; high-quality proompting; accountable ownership of reasoning in human–AI interaction.
	Quantitative	Faster construction of reliable ML models with balanced, unbiased datasets (e.g., with LearningML).
	Qualitative	Focused, planned, self-regulated and iterative proompting, evidenced by an explicit prompt-history log.
	Social	Integrating ML models into applications genuinely used by real third-party audiences.
**Stage #3 Quantum CT**	General profile	Exceptional mastery of Quantum CT; reasoning through superposition, entanglement, interference, coherence and measurement; designing transformations through gates and circuits; connecting quantum rules with computational structures; epistemic maturity and flexible movement across concepts, practices and perspectives.
	Quantitative	Solving quantum gates and quantum puzzles typically associated with high school already in middle school.
	Qualitative	Conscious shifting between quantum and classical mindsets; access to quantum picturalism or symbolic algebra beyond mere metaphor; greater ease with quantum paradoxes.
	Social	Communicating quantum ideas to others; extrapolating quantum principles to group leadership and organizational management (Zohar, 2022a, 2022b).

## 4. Conclusions, Implications and Further Work

This theoretical article has addressed two closely related aims. First, it has further developed the 3-stage framework of computational thinking (CT) (Román-González et al., 2025), according to which CT can be understood as a dynamic psychological construct that evolves in parallel with the development of Computer Science. Second, it has proposed an updated definition of computational talent by extending it beyond its initial rule-based formulation toward data-driven and quantum forms of computational reasoning. In this sense, the article treats early or accelerated mastery of programming as a relevant indicator within the first stage, while defining computational talent more broadly as a domain-specific, historically situated, and progressively developing capacity to perform at an outstanding level in computationally mediated problem-solving and idea-expression tasks.

More specifically, the article has distinguished three broad stages in the evolution of CT and computational talent. In Stage #1, rule-based CT, computational talent is mainly expressed through the fluent use of procedural programming structures, abstraction, debugging, modularization, and the capacity to move from visual block-based programming to more formal text-based environments. In Stage #2, data-driven CT, computational talent expands toward the ability to reason with data, build and evaluate AI/ML models, interpret probabilistic outputs, identify bias, engage in high-quality prompting or proompting, and preserve epistemic responsibility in human–AI interaction. In Stage #3, quantum CT, computational talent is tentatively conceptualized as quantum computational fluency: the ability to think with qubits, superposition, entanglement, interference, coherence, gates, circuits, measurement, and probabilistic outcomes, while evaluating the possibilities and limits of quantum technologies with scientific caution.

Within each of these stages, talent was distinguished from mere proficiency through a triple criterion—quantitative, qualitative, and social—drawn from the classical models of educational intervention for gifted students: acceleration, enrichment, and grouping, respectively (Tourón, 2024). This criterion supplies an explicit and consistent analytical lens across the three stages: quantitative acceleration from block-based to text-based programming in Stage #1; qualitative depth of proompting together with the social integration of AI/ML models into shared applications in Stage #2; and the quantitative, qualitative, and social manifestations of quantum reasoning explored in Stage #3. Table 1 synthesizes the evolution of CT itself, organizing the three stages according to their computing paradigm, programming paradigm, educational tools, underlying logic, philosophical orientation, and CT concepts, practices, and perspectives. Table 2, on this same analytical basis, synthesizes the corresponding profile of computational talent and its quantitative, qualitative, and social expressions in each stage.

The main contribution of this article is therefore conceptual: it proposes that computational talent constitutes a dynamic construct whose meaning must be periodically revisited as the computational paradigms that shape Computer Science Education change. A student considered computationally talented within the rule-based paradigm may show excellence in algorithmic design, abstraction, and programming fluency; in the current educational landscape, however, this definition falls short. The emergence of AI, Generative AI, and quantum computing calls for a broader conception of computational talent, one that encompasses deductive and procedural reasoning together with inductive, probabilistic, evaluative, relational, and epistemically cautious forms of thought.

This proposal has several educational implications. First, it suggests that the identification of computationally talented students should be broadened. Traditional indicators, such as programming acceleration or performance in rule-based tasks, remain valuable, but they should be complemented with evidence of model-oriented reasoning, critical evaluation of AI outputs, responsible use of GenAI, and, in emerging contexts, quantum literacy. In the GenAI era, particular caution is warranted: the apparent quality of a final product may no longer be a sufficient indicator of talent, since AI systems can substantially contribute to polished outputs. Assessment should therefore place greater emphasis on process-based evidence, including problem framing, prompt design, dataset construction, model validation, error interpretation, justification of revisions, and ownership of reasoning.

Second, this framework has curricular implications. If CT is evolving from rule-based to data-driven and quantum forms, Computer Science Education should extend beyond introductory programming. Programming remains essential, and should be progressively integrated with AI literacy, data literacy, critical algorithmic awareness, GenAI interaction, and introductory quantum literacy. This calls for coherent learning progressions in which students first learn to make procedures explicit, then to reason with data-driven systems, and later approach non-classical forms of information processing, rather than adding disconnected technological topics to already overloaded curricula. As Table 1 and Table 2 jointly illustrate, this progression involves a change in tools together with a transformation in the concepts, practices, perspectives, and forms of talent associated with each computational paradigm.

Third, the article also has implications for gifted and talented education. Computational talent should be understood as a specific talent domain within Computer Science Education, distinct from a manifestation of general high intellectual ability. This distinction matters because computationally talented students may require differentiated opportunities: accelerated programming pathways, advanced abstraction tasks, AI/ML projects, critical GenAI challenges, mentorship, participation in computational communities, and, where appropriate, early exposure to quantum concepts through accessible, visual, game-based, or simulation-based environments. Such opportunities should cultivate technical performance alongside agency, creativity, epistemic vigilance, ethical responsibility, and intellectual humility.

Fourth, the proposed framework invites a deeper pedagogical reflection. The evolution from rule-based to data-driven and quantum CT constitutes both a technical transition and a shift in how learners understand knowledge, uncertainty, and human agency. Rule-based CT emphasizes explicit procedure and deterministic execution. Data-driven CT introduces uncertainty, approximation, bias, and model evaluation. Quantum CT further challenges learners to reason about relationality, measurement, probability, and the limits of direct observation. As a result, the development of computational talent should orient itself toward helping learners understand how computational models shape the way reality is represented, interpreted, and transformed, beyond the mere production of efficient computational solutions. In particular, while existing studies provide empirical support for the educational feasibility of AI/ML, structured prompting, and quantum literacy assessment, their use as indicators of computational talent remains a theoretical extension that requires further psychometric, longitudinal, and classroom-based validation.

Nevertheless, the proposal advanced in this article should be interpreted with caution. As stated at the outset, this manuscript is conceived as a theoretical essay: its purpose is to articulate a coherent conceptual framework and to provoke reflection and discussion within the field, not to report new empirical findings, and the 3-stage CT framework accordingly remains theoretically grounded rather than empirically validated. Stage #1 is relatively mature, with consolidated tools, practices, and assessment approaches. Stage #2 is rapidly expanding, especially due to AI and GenAI, though its educational and psychometric implications are still being clarified. Stage #3 is the most emergent, since K-12 quantum education remains in an early phase. Further research should accordingly move from conceptual proposal to empirical development, examining the framework from its historical, developmental, pedagogical, and conceptual dimensions.

Research should deepen the theoretical and empirical characterization of Stage #2 and Stage #3, since data-driven CT and quantum CT remain less consolidated than rule-based CT. This requires developing and validating assessment tools capable of capturing the specific forms of reasoning involved in AI/ML, Generative AI, model evaluation, probabilistic inference, quantum literacy, and Quantum CT. Such tools should be pedagogically meaningful and developmentally appropriate, so that they can contribute not only to measuring performance, but also to identifying computational potential from early ages.

Also, future studies should design, implement, and evaluate educational programs that translate the three-stage framework into classroom practice. These programs should offer progressive pathways from Stage #1 to Stage #2 and Stage #3: beginning with procedural programming, abstraction, debugging, and modularization; expanding toward data-driven reasoning, AI literacy, critical prompting, and model evaluation; and gradually introducing quantum concepts through visual, playful, simulation-based, and scaffolded approaches. In parallel, it will be necessary to collect and synthesize scientific evidence on existing CT programs that incorporate AI, in order to understand which pedagogical designs, tools, tasks, and forms of teacher mediation are most effective in fostering computational reasoning.

Finally, further research should examine the developmental trajectory of computational talent. This includes studying whether early talent in programming predicts later performance in AI-related or quantum computational tasks, how computational potential can be detected in young learners, and which variables influence its growth over time. These variables may include cognitive factors, such as abstraction, logical reasoning, spatial ability, creativity, metacognition, and tolerance of uncertainty; motivational factors, such as interest, persistence, curiosity, and self-efficacy; pedagogical factors, such as access to challenging tasks, feedback, mentoring, and differentiated pathways; and contextual factors, such as technological access, teacher preparation, gender stereotypes, and sociocultural opportunities. From this perspective, the central educational challenge consists of creating the conditions under which computational talent can be recognized, nurtured, and responsibly developed, well beyond its mere identification.

In terms of priorities, the most immediate task is to develop assessment criteria for Stage #2, especially for AI/ML model evaluation, prompting quality, bias detection, and ownership of reasoning. A longer-term priority is to refine and validate Stage #3 educational proposals, since quantum CT remains more exploratory and requires further empirical evidence.

In conclusion, extending the definition of computational talent constitutes an educational necessity derived from the transformation of computation itself, rather than a merely terminological task. If Computer Science evolves, the forms of thought, creativity, judgment, and excellence associated with it must also be reconsidered. Computationally talented students in the coming decades will be those who can reason across computational paradigms, well beyond programming efficiently: designing explicit procedures, evaluating data-driven systems, interacting critically with AI, and approaching quantum technologies with rigor, imagination, and caution. In this broader sense, computational talent may be understood as a developing form of computational agency: the capacity to think, create, evaluate, and act responsibly in a world increasingly shaped by classical, artificial, and quantum forms of computation.

## Figures and Tables

**Table 1:** CT framework evolution and expansion across three stages.

Stage	Aspect	Description
Stage #1 Rule-based CT	Computing paradigm	Classical binary computing.
	Programming paradigm	Rule-based/procedural programming.
	K-12 educational tools	Scratch, App Inventor, Snap!, Blockly, Code.org.
	Type of logic involved	Deductive and imperative logic (top-down).
	Philosophical paradigm	Deterministic.
	CT concepts	Sequences; loops; events; parallelism; conditionals; operators; data.
	CT practices	Incremental and iterative design; testing and debugging; reusing and remixing; abstracting and modularizing.
	CT perspectives	Expressing; connecting; questioning.
Stage #2 Data-driven CT	Computing paradigm	AI-based computing; data-driven computing; Machine Learning and Generative AI systems.
	Programming paradigm	Model construction and validation; natural language programming; prompting/proompting.
	K-12 educational tools	LearningML; Machine Learning for Kids; App Planner; CoRemix; GenAI tools such as ChatGPT, Copilot, Gemini, Midjourney, or Stable Diffusion, when pedagogically scaffolded.
	Type of logic involved	Inductive and probabilistic logic (bottom-up); iterative human–AI interaction.
	Philosophical paradigm	Probabilistic; data-driven; model-based; uncertainty-oriented.
	CT concepts	Classification; prediction; generation.
	CT practices	Training; validating; testing; prompting/proompting; refining AI outputs; evaluating model behaviour.
	CT perspectives	Evaluating; epistemic vigilance; responsible human–AI agency.
Stage #3 Quantum CT	Computing paradigm	Quantum computing; quantum information processing; non-binary computational paradigm based on qubits, quantum states, superposition, entanglement, interference, and measurement.
	Programming paradigm	Quantum programming; circuit-based programming; design of quantum gates, circuits, simulations, and algorithms; interpretation of repeated measurements and probabilistic outputs.
	K-12 educational tools	Quantum games and serious games; simulators; unplugged tools; visual and puzzle-based tools; block-based or scaffolded environments; QScratch, QPlayLearn, Qubit by Qubit, Quarks Interactive, Qiskit 2.0, quantum tic-tac-toe, quantum battleship, and other pedagogically mediated quantum platforms.
	Type of logic involved	Non-classical, probabilistic, and relational logic; reasoning through superposition, entanglement, interference, coherence, reversibility, measurement, and uncertainty.
	Philosophical paradigm	Quantum; holistic and epistemic; uncertainty-oriented; relational; focused on the interdependence between information, physical reality, observation, and measurement.
	CT concepts	Qubits; quantum states; superposition; entanglement; measurement; interference; coherence; quantum gates; circuits; quantum algorithms; teleportation; tunnelling; reversibility; quantum advantage; probability; encryption and cryptography.
	CT practices	Modelling quantum states; predicting probabilistic outcomes; designing and interpreting circuits; running simulations; comparing expected and observed results; testing quantum procedures; reasoning with multiple representations; preserving coherence; intentionally collapsing through measurement; evaluating quantum outputs with scientific caution.
	CT perspectives	Assuming uncertainty; recognizing the role of measurement; understanding the limits of direct observation; interpreting information as physically instantiated; recognizing relational structures; unity within diversity; epistemic caution; transcendent thinking about knowledge, reality, and meaning.

## Data Availability

No new empirical data were created in this study. The sources analyzed are publicly available and are cited in the reference list.

## Notes

1 https://scratch.mit.edu/ (accessed on 1 May 2026).

2 https://appinventor.mit.edu/ (accessed on 1 May 2026).

3 https://snap.berkeley.edu/ (accessed on 1 May 2026).

4 https://learningml.org/ (accessed on 1 May 2026).

5 https://machinelearningforkids.co.uk/ (accessed on 1 May 2026).

6 https://www.quarksinteractive.com/ (accessed on 1 May 2026).

7 https://www.qubitbyqubit.org/ (accessed on 1 May 2026).

8 https://qplaylearn.com/ (accessed on 1 May 2026).

9 https://marcosrv-ull.github.io/QScratch (accessed on 1 May 2026).

## Abbreviations

The following abbreviations are used in this manuscript:

AI	Artificial Intelligence
AI/ML	Artificial Intelligence/Machine Learning
CS	Computer Science
CT	Computational Thinking
GenAI	Generative Artificial Intelligence
K-12	Kindergarten to Grade 12
ML	Machine Learning
QC	Quantum Computing
RAG	Retrieval-Augmented Generation
STEM	Science, Technology, Engineering, and Mathematics

## References

[B1-behavsci-16-01278] Beardow C., Stappers P. J. (2026). Towards metaphors for quantum computational thinking. EPJ Quantum Technology.

[B2-behavsci-16-01278] Bondani M., Amabile A., Malgieri M., Marzoli I., Nazzaro M., Hamma A. (2026). QTris: A new game for teaching quantum physics. Journal of Physics: Conference Series.

[B3-behavsci-16-01278] Brackmann C. P., Román-González M., Robles G., Moreno-León J., Casali A., Barone D. (2017). Development of computational thinking skills through unplugged activities in primary school. 12th Workshop on Primary and Secondary Computing Education.

[B4-behavsci-16-01278] Brennan K., Resnick M. (2012). New frameworks for studying and assessing the development of computational thinking *[Paper presentation]*. Annual Meeting of the American Educational Research Association.

[B5-behavsci-16-01278] Brummelen J. V., Shen J. H., Patton E. W. (2019). The popstar, the poet, and the grinch: Relating artificial intelligence to the computational thinking framework with block-based coding. Proceedings of the international conference on computational thinking education 2019.

[B6-behavsci-16-01278] Chen Y., Yu X., Shen Y., Liu R., Sun L., Chen L. (2025). CoRemix: Supporting online learning in Scratch community with visual flowchart and generative AI. International Journal of Human–Computer Interaction.

[B7-behavsci-16-01278] Creely E. (2026). Generative AI to foster computational thinking in initial teacher education: A thematic literature review and model. Behavioral Sciences.

[B8-behavsci-16-01278] Dündar-Coecke S., Yeh L., Pothos E., Waseem M. H., Coecke B. (2026). Beyond symbolic algebra with quantum picturalism. Frontiers in Cognition.

[B9-behavsci-16-01278] Dündar-Coecke S., Yeh L., Puca C., Pfaendler S. M.-L., Waseem M. H., Cervoni T., Kissinger A., Gogioso S., Coecke B. (2023). Quantum picturalism: Learning quantum theory in high school. 2023 IEEE international conference on quantum computing and engineering (QCE).

[B10-behavsci-16-01278] Escanez-Exposito D., Rodríguez-Vega M., Rosa-Remedios C., Caballero-Gil P. (2025). QScratch: Introduction to quantum mechanics concepts through block-based programming. EPJ Quantum Technology.

[B11-behavsci-16-01278] Farooq U., Upadhyay K. (2025). Teaching quantum computing through lab-integrated learning: Bridging conceptual and computational understanding. arXiv.

[B12-behavsci-16-01278] Foti C., Anttila D., Maniscalco S., Chiofalo M. (2021). Quantum physics literacy aimed at K12 and the general public. Universe.

[B13-behavsci-16-01278] Gagné F. (2004). Transforming gifts into talents: The DMGT as a developmental theory. High Ability Studies.

[B14-behavsci-16-01278] Garcés M. G., Gómez Orzechowski L., Rodríguez Hernández J. F. (2025). Introducing quantum computing to high-school curricula: A global perspective (Version 1) [Preprint]. arXiv.

[B15-behavsci-16-01278] García-Peñalvo F. J., Llorens-Largo F., Vidal J. (2024). La nueva realidad de la educación ante los avances de la inteligencia artificial generativa [The new reality of education in light of advances in generative artificial intelligence]. RIED-Revista Iberoamericana de Educación a Distancia.

[B16-behavsci-16-01278] Grover S. (2024). Teaching AI to K-12 learners: Lessons, issues, and guidance. Proceedings of the 55th ACM technical symposium on computer science education V.1.

[B17-behavsci-16-01278] Guilbault K. M., Wang Y., McCormick K. M. (2025). Using ChatGPT in the secondary gifted classroom for personalized learning and mentoring. Gifted Child Today.

[B18-behavsci-16-01278] Halfhill J., Pace N. R., Peters O. W., Kashinski D. O. (2026). Advocating for quantum curricula in primary and secondary education programs. Discover Education.

[B19-behavsci-16-01278] Hsu H.-P. (2025). From programming to prompting: Developing computational thinking through large language model-based generative artificial intelligence. TechTrends.

[B20-behavsci-16-01278] Kim D. Y. J., Ravi P., Williams R., Yoo D. (2024). App planner: Utilizing generative AI in K-12 mobile app development education. Proceedings of the 23rd annual ACM interaction design and children conference.

[B21-behavsci-16-01278] Kong S.-C., Abelson H., Lai M., Kong S.-C., Abelson H. (2019). Introduction to computational thinking education. Computational thinking education.

[B22-behavsci-16-01278] Lubbe A., Marais E., Kruger D. (2025). Cultivating independent thinkers: The triad of artificial intelligence, Bloom’s taxonomy and critical thinking in assessment pedagogy. Education and Information Technologies.

[B23-behavsci-16-01278] Moreno-León J., Robles G., Román M., Rodríguez J. D. (2019). No es lo mismo: Un análisis de red de texto sobre definiciones de pensamiento computacional para estudiar su relación con la programación informática [Not the same: A text network analysis on computational thinking definitions to study its relationship with computer programming]. Revista Interuniversitaria de Investigación en Tecnología Educativa.

[B24-behavsci-16-01278] Moreno-León J., Romero-Organvídez D., Robles G., Benavides D. (2025). Software variability as a new dimension of computational thinking: An exploration. Proceedings of the 29th ACM international systems and software product line conference.

[B25-behavsci-16-01278] Moss M., Mehta G. (2026). Q’ Game: An interactive game developed for teaching quantum computing basics. 2026 IEEE 19th dallas circuits and systems conference (DCAS).

[B26-behavsci-16-01278] Nita L., Mazzoli Smith L., Chancellor N., Cramman H. (2023). The challenge and opportunities of quantum literacy for future education and transdisciplinary problem-solving. Research in Science & Technological Education.

[B27-behavsci-16-01278] Preiss D. D., Sternberg R. J. (2025). Human intelligence, creativity, and wisdom in the age of generative artificial intelligence. Creativity: Theories—Research—Applications.

[B28-behavsci-16-01278] Qian Y., Choi I. (2023). Tracing the essence: Ways to develop abstraction in computational thinking. Educational Technology Research and Development.

[B29-behavsci-16-01278] Repenning A., Grabowski S. (2023). Proompting is computational thinking. Proceedings of the IS-EUD 2023: 9th international symposium on end-user development.

[B30-behavsci-16-01278] Rindle M., Goecke B., Wilhelm O., Weiss S. (2026). Intelligent AI use: Towards a performance-based assessment of AI literacy [Preprint]. ResearchGate.

[B31-behavsci-16-01278] Robres A. Q., Lozano Blasco R. (2020). Modelos de inteligencia y altas capacidades: Una revisión descriptiva y comparativa [Models of intelligence and high abilities: A descriptive and comparative review]. Enseñanza & Teaching: Revista Interuniversitaria de Didáctica.

[B32-behavsci-16-01278] Rodríguez-Fernández M. I., Sternberg R. J. (2024). The search for meaning in the life of the gifted. Gifted Education International.

[B33-behavsci-16-01278] Rodríguez-García J. D., Moreno-León J., Román-González M., Robles G. (2019). Developing computational thinking at school with machine learning: An exploration. 2019 International Symposium on Computers in Education (SIIE).

[B34-behavsci-16-01278] Rodríguez-García J. D., Moreno-León J., Román-González M., Robles G. (2020). LearningML: A tool to foster computational thinking skills through practical artificial intelligence projects [LearningML: Una herramienta para fomentar las habilidades de pensamiento computacional mediante proyectos prácticos de inteligencia artificial]. Revista de Educación a Distancia (RED).

[B35-behavsci-16-01278] Román-González M., Moreno-León J., Robles G., Kong S.-C., Abelson H. (2019). Combining assessment tools for a comprehensive evaluation of computational thinking interventions. Computational thinking education.

[B36-behavsci-16-01278] Román-González M., Moreno-León J., Robles G., Kong S.-C., Abelson H. (2022). Toward a theory (and practice) of multiple computational thinkings. Computational thinking education in K–12.

[B37-behavsci-16-01278] Román-González M., Moreno-León J., Robles G., Rodríguez J. D. (2025). Extending the computational thinking framework towards quantum education [Preprint]. ResearchGate.

[B38-behavsci-16-01278] Román-González M., Pérez-González J.-C., Abelson H., Kong S.-C. (2024). Computational thinking assessment: A developmental approach. Computational thinking curricula in K–12.

[B39-behavsci-16-01278] Román-González M., Pérez-González J.-C., Moreno-León J., Robles G. (2018a). Can computational talent be detected? Predictive validity of the Computational Thinking Test. International Journal of Child-Computer Interaction.

[B40-behavsci-16-01278] Román-González M., Pérez-González J.-C., Moreno-León J., Robles G. (2018b). Extending the nomological network of computational thinking with non-cognitive factors. Computers in Human Behavior.

[B41-behavsci-16-01278] Siegle D. (2025). Using AI prompt engineering to improve gifted students’ questioning. Gifted Child Today.

[B42-behavsci-16-01278] Sternberg R. J. (2023). Giftedness does not reside within a person: Defining giftedness in society is a three-step process. Roeper Review.

[B43-behavsci-16-01278] Sternberg R. J. (2025). Reconsidering what it means to be “gifted” in the age of AI.

[B44-behavsci-16-01278] Sternberg R. J. (2026). Does AI increase cognitive abilities, decrease them, or a little bit of each? And what are its implications for identification and development of the gifted?. Frontiers in Education.

[B45-behavsci-16-01278] Subotnik R. F., Olszewski-Kubilius P., Worrell F. C. (2011). Rethinking giftedness and gifted education: A proposed direction forward based on psychological science. Psychological Science in the Public Interest.

[B46-behavsci-16-01278] Tedre M. (2022). Computational thinking 2.0. Proceedings of the 17th workshop in primary and secondary computing education.

[B47-behavsci-16-01278] Tourón J. (2024). Navegando hacia el talento: Porque el talento que no se cultiva, se pierde *[Navigating toward talent: Because talent that is not cultivated is lost]*.

[B48-behavsci-16-01278] Wing J. M. (2006). Computational thinking. Communications of the ACM.

[B49-behavsci-16-01278] Xenakis A., Avramouli M., Sabani M., Savvas I., Chaikalis C., Theodoropoulou K. (2023). Quantum serious games to boost quantum literacy within computational thinking 2.0 framework. 2023 IEEE global engineering education conference (EDUCON).

[B50-behavsci-16-01278] Yusuf A., Pervin N., Román-González M. (2024a). Generative AI and the future of higher education: A threat to academic integrity or reformation? Evidence from multicultural perspectives. International Journal of Educational Technology in Higher Education.

[B51-behavsci-16-01278] Yusuf A., Román-González M., Atan N. A., Behera S. K., Md Noor N. (2025). Development of a Quantum Literacy Test for K-12 students: An extension of the computational thinking framework. Education Sciences.

[B52-behavsci-16-01278] Yusuf A., Román-González M., Behera S. K. (2024b). Quantum computing in K-12 education: An early systematic review. Computer Science Education.

[B53-behavsci-16-01278] Zhong M., Duan S., Wang L. (2026). Integrating computational thinking into K-12 artificial intelligence education. Education Sciences.

[B54-behavsci-16-01278] Zohar D. (2022a). Twelve principles of quantum leadership. Zero distance.

[B55-behavsci-16-01278] Zohar D. (2022b). What is quantum management?. Zero distance.

